# Comparison of lung ultrasound scoring systems for the prognosis of COVID‐19 in the emergency department: An international prospective cohort study

**DOI:** 10.1002/ajum.12364

**Published:** 2023-10-29

**Authors:** Peter J Snelling, Philip Jones, Rory Connolly, Tomislav Jelic, Dan Mirsch, Frank Myslik, Luke Phillips, Gabriel Blecher, the COVID LUS Study Group, Madeleine Champagne, Tarek Elsayed, Brooke Lerner

**Affiliations:** ^1^ Department of Emergency Medicine Gold Coast University Hospital Southport Queensland Australia; ^2^ School of Medicine and Dentistry Griffith University Southport Queensland Australia; ^3^ Sonography Innovation and Research Group Southport Queensland Australia; ^4^ Department of Emergency Medicine University of Ottawa Ottawa Ontario Canada; ^5^ Department of Emergency Medicine University of Manitoba Winnipeg Manitoba Canada; ^6^ Department of Emergency Medicine University at Buffalo Buffalo New York USA; ^7^ Division of Emergency Medicine Western University London Ontario Canada; ^8^ Department of Emergency Medicine Alfred Hospital Melbourne Victoria Australia; ^9^ Department of Epidemiology and Preventative Medicine Monash University Melbourne Victoria Australia; ^10^ Emergency Services, Peninsula Health Frankston Victoria Australia; ^11^ Peninsula Clinical School Monash University Melbourne Victoria Australia

**Keywords:** COVID‐19, lung, point‐of‐care ultrasound, prognosis, ultrasonography

## Abstract

**Purpose:**

The purpose of this study was to evaluate whether the lung ultrasound (LUS) scores applied to an international cohort of patients presenting to the emergency department (ED) with suspected COVID‐19, and subsequently admitted with proven disease, could prognosticate clinical outcomes.

**Methods:**

This was an international, multicentre, prospective, observational cohort study of patients who received LUS and were followed for the composite primary outcome of intubation, intensive care unit (ICU) admission or death. LUS scores were later applied including two 12‐zone protocols (‘de Alencar score’ and ‘CLUE score’), a 12‐zone protocol with lung and pleural findings (‘Ji score’) and an 11‐zone protocol (‘Tung‐Chen score’). The primary analysis comprised logistic regression modelling of the composite primary outcome, with the LUS scores analysed individually as predictor variables.

**Results:**

Between April 2020 to April 2022, 129 patients with COVID‐19 had LUS performed according to the protocol and 24 (18.6%) met the composite primary endpoint. No association was seen between the LUS score and the composite primary end point for the de Alencar score [odds ratio (OR) = 1.04; 95% confidence interval (CI): 0.97–1.11; P = 0.29], the CLUE score (OR = 1.03; 95% CI: 0.96–1.10; P = 0.40), the Ji score (OR = 1.02; 95% CI: 0.97–1.07; P = 0.40) or the Tung‐Chen score (OR = 1.02; 95% CI: 0.97–1.08).

**Discussion:**

Compared to these earlier studies performed at the start of the pandemic, the negative outcome of our study could reflect the changing scenario of the COVID‐19 pandemic, including patient, disease, and system factors. The analysis suggests that the study may have been underpowered to detect a weaker association between a LUS score and the primary outcome.

**Conclusion:**

In an international cohort of adult patients presenting to the ED with suspected COVID‐19 disease who had LUS performed and were subsequently admitted to hospital, LUS severity scores did not prognosticate the need for invasive ventilation, ICU admission or death.

## Introduction

The SARS‐CoV‐2 (COVID‐19) global pandemic has had a profound impact, resulting in an estimated 18 million deaths worldwide by the end of 2021.^1^ Point‐of‐care ultrasound in the emergency department (ED) has been valuable in many parts of the world during the pandemic as it is readily available, easy to sanitise and portable, and can immediately supply clinical information.^2^, ^3^ In particular, the utility of lung ultrasound (LUS) for the diagnosis and prognosis of COVID‐19 pneumonia for patients presenting to the ED has been explored.^4^, ^5^ However, while the LUS scores have been developed for assessing the severity of COVID‐19,^6^, ^7^, ^8^, ^9^ there have not been any comparative studies for their prognostic value on an international cohort of patients.

Lung ultrasound is known to be more sensitive for the detection of lung pathology than plain X‐ray and is comparable to computed tomography (CT), particularly for peripheral or pleural lesions.^10^ Therefore, LUS is thought to be ideal for the evaluation of COVID‐19 pneumonia, which mainly affects the peripheral lung parenchyma, interstitium and pleura.^11^ It has grown in favour during the pandemic due to its relative availability, portability, rapidity and affordability, in comparison with other imaging modalities, such as CT.^12^ Although chest CT gives arguably more comprehensive images, the infection control requirements when scanning COVID‐19‐positive patients could lead to decreased efficiency of the CT scanner throughput and increased risk of nosocomial infection. The main utility of LUS for COVID‐19 has been proposed for the diagnosis and prognosis of the disease, with the LUS scores devised to risk stratify the severity of lung disease.^4^, ^5^

Lung ultrasound has been useful for the evaluation of COVID‐19 pneumonia, given that most of the findings are peripheral based.^13^, ^14^ Unfortunately, the diagnostic utility of LUS for COVID‐19 has been limited by non‐specific findings, which overlap with other viral respiratory infections.^5^, ^15^ The LUS scores for the stratification of COVID‐19 severity have been devised, which incorporate the main elements of lung findings (B‐lines, subpleural consolidation and lung hepatisation) and pleural findings (absence of lung sliding, pleural line abnormalities and pleural effusions).^6^, ^7^, ^8^, ^9^ These LUS scores have been used to stratify the severity of disease, to assist with disposition and prognostication.

Although LUS is unlikely to replace nucleic acid [reverse transcriptase polymerase chain reaction (RT‐PCR)] testing for definitive diagnosis,^15^, ^16^ it may have utility in predicting clinical deterioration and indicating the need for ventilatory support. If proven to accurately predict clinical deterioration, LUS findings could be integrated with clinical findings at the time of ED presentation, to triage patients to either outpatient management (low‐risk features), ward admission (moderate‐risk features) or intensive care unit (ICU) admission (high‐risk features).^17^ This could potentially impact patient outcomes, resource allocation and departmental flow, particularly in times of crisis and overcrowding.

The objective of this study was to evaluate whether the LUS scores of an international cohort of patients presenting to the ED with suspected COVID‐19 could prognosticate invasive ventilation, ICU admission or death, when admitted to hospital with proven disease.

## Methods and materials

### Study design

This was an international, multicentre, prospective, observational cohort study of patients presenting to ED who were admitted to hospital with a positive RT‐PCR test for COVID‐19, received LUS and were followed up for the composite outcome of intubation, ICU or high‐dependency unit (HDU) admission or death. The study was approved by the Monash Health Human Research Ethics Committee (RES‐20‐0000‐284A) with each international site obtaining local approval. The study was prospectively registered with the Australian and New Zealand Clinical Trials Registry (ACTRN12620000734965) and has been reported according to the Strengthening the Reporting of Observational Studies in Epidemiology statement.^18^

### Study setting and population

The study was conducted across participating international sites, which were tertiary referral centres with an annual census of adult patients ranging between 70,000 and 100,000 located in the United States, Canada and Australia. The sites included the Buffalo General Hospital (Buffalo, NY, USA), Ottawa Hospital (Ottawa, Ontario, Canada), Health Sciences Centre (Winnipeg, Manitoba, Canada), London Health Sciences Centre (London, Ontario, Canada), Alfred Hospital (Melbourne, Victoria, Australia) and Gold Coast University Hospital (Gold Coast, Queensland, Australia). The study population included patients aged 18 years or older who presented to the ED and were admitted with COVID‐19 confirmed on RT‐PCR testing. Patients were excluded if they were deemed to be for palliation, had pre‐existing lung disease (pneumonectomy, malignancy, pleurodesis and interstitial lung disease) or tested negative for COVID‐19.

### Study procedures

Sites asynchronously commenced in the study, with patients then recruited as a convenience sample at each site. The clinicians enrolling patients across all sites included both emergency physicians (n = 42) and senior trainees, that is trainees in their final years of training (n = 16), who had been credentialed within their own institute to perform LUS, which included at least 50 prior LUS scans. These clinicians screened potentially eligible patients when working on a clinical shift. Patients were approached and provided with written information regarding the study with the opportunity to ask questions before electronic consent was obtained, as required by the local ethics committee. Machines were disinfected, and LUS was performed in full personal protective equipment as per each participating site's local policy. Clinicians performing the LUS were blinded to any prior imaging, including X‐ray or CT. Findings were documented in the medical records, and data were later entered into the REDCap® database (Vanderbilt University, Nashville, TN, USA). Images were quality‐assured at each site by the ultrasound site lead. LUS findings were entered as per findings in each zone, with the LUS scores later calculated by the lead author who was blinded to their outcomes.

### Intervention

**Figure 1:**
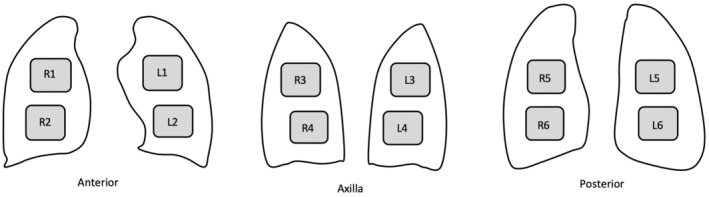
Lung ultrasound scanning protocol.

Utilising a point‐of‐care ultrasound machine, a 12‐zone LUS protocol was performed^19^, ^20^ (Figure 1), unless the patient could only be scanned anteriorly, using a curvilinear or phased array probe with a ‘lung’ preset (artefact reduction features, such as tissue harmonic imaging or cross‐beam enhancement, disabled), a depth set to at least 8–10 cm and the focus set to the pleural line. A 3‐ to 4‐second cine loop was obtained in each region. Additional images could be recorded, including the use of a high‐frequency linear probe, at the discretion of the ED clinician. Clinicians performing the scan documented pleural findings, including absence of lung sliding, pleural line abnormalities and pleural effusions, and lung findings, including B‐lines (number/distribution) and consolidation (subpleural < 1 cm, >1 cm or hepatisation). The LUS scores were calculated according to the previously published systems, selected by the authors to represent the main variety of protocols, including two 12‐zone protocols giving 0–36 total scores (referred to as the ‘de Alencar score’^9^ and the ‘CLUE score’^6^), 12‐zone protocol with lung and pleural findings giving a 0–60 total score (‘Ji score’^8^) and 11‐zone protocol giving a 0–33 total score (‘Tung‐Chen score’^7^). In all cases, higher scores denote more significant changes.

### Outcomes

The primary composite outcome was the need for endotracheal intubation and ventilation, ICU or HDU (hereafter combined as ICU) admission from ED, or in‐hospital mortality. Secondary outcomes included the components of the primary composite outcome as well as the composite end point of intubation, ICU admission or in‐hospital mortality within 72 h of enrolment. Other secondary outcome measures included the correlation of the LUS scores with level of respiratory support and fraction of inspired oxygen at enrolment and hospital length of stay. Clinical information was obtained when LUS was performed, including days since symptom onset, comorbidities, COVID‐19 vaccination status, heart rate, respiratory rate, blood pressure, oxygen saturation and any oxygen delivery (mode/fraction of inspired oxygen). Additionally, findings of any chest X‐ray or CT scan performed on ED presentation were recorded, as reported by a radiologist.

### Sample size

Sample size calculations assumed that the primary composite outcome of endotracheal intubation, ICU admission or death would occur in around a third of admissions based on data from a prior study,^21^ with the assumption that disease patterns would remain constant (e.g. virulence of the virus). A sample size of 1000 COVID‐19 admissions was planned, to allow the true prevalence of the composite end point to be estimated to within three percentage points either side of the estimated prevalence, at a 95% confidence interval (CI) level. Under these assumptions, the study would have greater than 80% power if a participant with a high LUS score [defined as mean + one standard deviation (SD)] had a true probability of 0.374 or greater of meeting the primary end point (Table S1).

### Data analysis

The primary analysis comprised logistic regression modelling of intubation, ICU admission and death, with the LUS scores analysed individually as predictor variables. An association between the LUS score and the composite end point was determined if the 95% CI for the associated regression coefficient did not cross the null. Explained variability was described using McFadden's pseudo‐R^2^. Continuous data were reported as mean (SD) or median [interquartile range (IQR)]. Categorical data were presented as frequency and percentage.

Associations between the primary composite end point and patient characteristics, chest X‐ray and CT findings were described as odds ratios (OR), with statistical significance testing performed using the chi‐squared test for categorical predictors and logistic regression for continuous predictors. Agreement between the LUS scores was enumerated using pairwise Pearson coefficients, with agreement by lung zone analysed with linear weighted kappa. Level of respiratory support, fraction of inspired oxygen and hospital length of stay were analysed using ordered logistic regression, linear regression and negative binomial regression, respectively.

Missing data were assumed to be missing at random. For regression analysis modelling, missing data were multiply‐imputed using ordered logistic regression, with LUS values for other lung zones for that participant as predictors. Complete case analysis and single imputation using the mean of non‐missing lung zone scores for that participant were also reported. Formal adjustment of CIs for multiplicity was not performed. Analyses were performed using Stata V17.0 (StataCorp, College Station, TX, USA).

## Results

### Patient characteristics

Between 21 April 2020 and 14 April 2022, 134 patients underwent LUS according to the protocol. Five participants did not have a positive RT‐PCR for COVID‐19 and were excluded from the analysis. Of the remaining 129, 24 met the composite primary end point, including 11 who required intubation and ventilation, 14 who required ICU admission and 10 who died (end points not exclusive). Baseline patient characteristics by primary outcome are summarised in Table 1. Higher estimated FiO2 (OR = 104; 95% CI: 12.4–887; P < 0.001), higher respiratory rate (OR = 1.10; 95% CI: 1.03–1.17; P = 0.005) and presence of fever (37% vs. 63%; OR = 2.82; 95% CI: 1.13–7.05; P = 0.023) at enrolment were associated with the primary outcome. Of recorded comorbidities, participants with chronic respiratory disease (10% vs. 29%; OR = 3.91; 95% CI: 1.34–8.47; P = 0.010), diabetes mellitus (23% vs. 50%; OR = 3.38; 95% CI: 1.34–8.47; P = 0.007) and hypertension (35% vs. 67%; OR = 3.68; 95% CI: 1.44–9.39; P = 0.005) were more likely to meet the primary outcome, whereas participants with no comorbidities (33% vs. 4%; OR = 0.09; 95% CI: 0.01–0.67; P = 0.004) were less likely to reach this end point. COVID‐19 vaccination data were missing for 28% of participants, so we were unable to provide an interpretation of any association.

**Table 1:** Patient characteristics by composite outcome of death, ICU or HDU admission, or intubation.

Characteristic	No death, ICU or HDU admission, or intubation (N = 105)	Death, ICU or HDU admission, or intubation (N = 24)	Overall (N = 129)	Odds ratio (95% CI)	P‐value
Age	57 (44.5–67.5)	66 (55.5–74)	59 (46–68)	1.02 (1.00–1.06)	0.066
Age greater than 50	68 (65%)	19 (79%)	87 (67%)	2.07 (0.71–5.99)	0.17
Gender – male	64 (61%)	14 (58%)	78 (60%)	0.90 (0.36–2.21)	0.81
Oxygen saturation	95 (93–97)	94 (92–96)	95 (93–96)	0.91 (0.79–1.05)	0.19
Estimated FiO2	0.28 (0.21–0.36)	0.44 (0.3–0.875)	0.28 (0.21–0.36)	104 (12.4–887)	<0.001
Respiratory rate (breaths per minute)	24 (18–28)	29 (24–32.5)	24 (20–30)	1.10 (1.03–1.17)	0.005
Systolic BP (mmHg)	128 (113–140)	131.5 (119.5–147)	128 (113–141)	1.01 (0.99–1.04)	0.18
Diastolic BP (mmHg)	78 (69–86)	79.5 (64.5–88)	78 (68–86)	1.00 (0.97–1.03)	0.91
Heart rate (beats per minute)	94 (84–105)	98 (86–109)	95 (84–106)	1.01 (0.99–1.03)	0.49
Fever	39 (37%)	15 (63%)	54 (42%)	2.82 (1.13–7.05)	0.023
Days of symptoms	7 (5.5–10)	6.5 (4.5–9)	7 (5–10)	0.95 (0.85–1.07)	0.40
Time period of recruitment^a^ ^s^					
Late 2020	10 (10%)	4 (17%)	14 (11%)	–	0.51
Early 2021	77 (73%)	17 (71%)	94 (73%)	–	
Late 2021	12 (11%)	3 (13%)	15 (12%)	–	
Early 2022	6 (6%)	0 (0%)	6 (5%)	–	
Site					
London Health Sciences Centre	52 (50%)	5 (21%)	57 (44%)	–	
Buffalo General Hospital	26 (25%)	7 (29%)	33 (26%)	–	
The Ottawa Hospital	12 (11%)	5 (21%)	17 (13%)	–	
Alfred Health	9 (9%)	3 (13%)	12 (9%)	–	
Health Sciences Centre	4 (4%)	4 (17%)	8 (6%)	–	
Gold Coast University Hospital	2 (2%)	0 (0%)	2 (2%)	–	0.050
COVID‐19 vaccination received^b^					
No vaccination	69 (85%)	10 (83%)	79 (85%)	–	0.88
Partial vaccination	8 (10%)	1 (8%)	9 (10%)	1.72 (0.17–17.0)	
Full vaccination	4 (5%)	1 (8%)	5 (5%)	0.86 (0.10–7.65)	
Patient comorbidities					
No comorbidities	35 (33%)	1 (4%)	36 (28%)	0.09 (0.01–0.67)	0.004
Active cancer	3 (3%)	1 (4%)	4 (3%)	1.48 (0.15–14.9)	0.74
Chronic respiratory disease	10 (10%)	7 (29%)	17 (13%)	3.91 (1.31–11.7)	0.010
Cigarette smoking	6 (6%)	1 (4%)	7 (5%)	0.72 (0.08–6.25)	0.76
Diabetes mellitus	24 (23%)	12 (50%)	36 (28%)	3.38 (1.34–8.47)	0.007
Heart failure	1 (1%)	2 (8%)	3 (2%)	9.45 (0.82–108.9)	0.030
Other chronic cardiac disease	9 (9%)	2 (8%)	11 (9%)	0.97 (0.20–4.81)	0.97
Hypertension	37 (35%)	16 (67%)	53 (41%)	3.68 (1.44–9.39)	0.005
Immunosuppressive medication	4 (4%)	1 (4%)	5 (4%)	1.10 (0.12–10.3)	0.94
Obesity	18 (17%)	6 (25%)	24 (19%)	1.61 (0.56–4.62)	0.37
Other comorbidities	27 (26%)	14 (58%)	41 (32%)	4.04 (1.61–10.2)	0.002

Data reported as median (IQR) for continuous data and number (percentage) for categorical data.

BP, blood pressure; CI, confidence interval; FiO2, fraction of inspired oxygen; HDU, high‐dependency unit; ICU, intensive care unit; mmHg, millimetres of mercury.

^a^ Early: January to June, Late: July to December of the specified year.

^b^ COVID‐19 vaccination data missing for 24 patients in the ‘No death, ICU or HDU admission or intubation’ group and for 12 patients in the ‘Death, ICU or HDU admission or intubation’ group.

### Primary outcome

**Figure 2:**
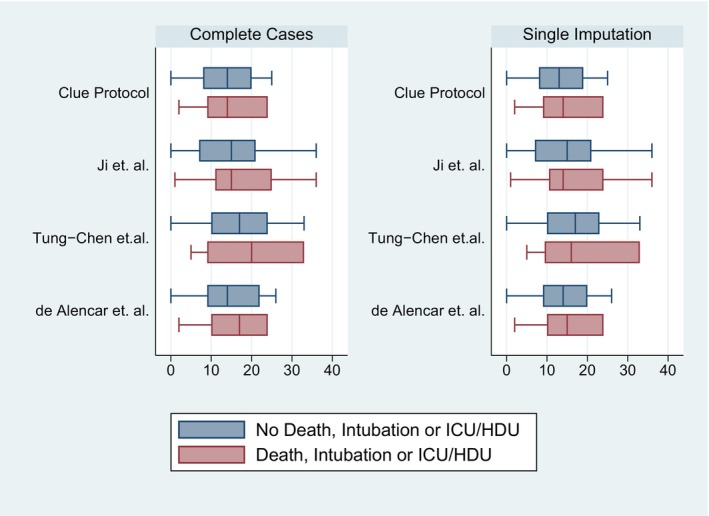
Lung ultrasound score by composite outcome of death, intubation or ICU/HDU admission; complete cases and single imputation by mean score. HDU, high‐dependency unit; ICU, intensive care unit.

Complete data were available for all lung zones for 118 participants, including 19 who met the primary outcome and 99 who did not. Summary statistics for the de Alencar, CLUE, Ji and Tung‐Chen scores are shown in Table 2 and Figure 2, including complete case and single imputation analyses. Of the 11 participants, eight were with missing data only from the posterior zones (R5, R6, L5 or L6) and the rest with missing data from the posterior and lateral zones, allowing robust multiple imputation for these participants.

**Table 2:** Summary statistics by COVID‐19 lung ultrasound score; complete cases and single imputation by mean score.

Score	No death, ICU or HDU admission or intubation			Death, ICU or HDU admission or intubation			Overall		
	N	Mean	Standard deviation	N	Mean	Standard deviation	N	Mean	Standard deviation
De Alencar score									
Complete cases	99	14.2	7.5	19	16.3	7.3	118	14.5	7.46
Single imputation – mean score	105	14.0	7.4	23	16.0	7.1	128	14.4	7.35
CLUE score									
Complete cases	99	13.1	7.2	19	14.8	7.2	118	13.4	7.24
Single imputation – mean score	105	13.0	7.2	23	14.6	7.1	128	13.3	7.16
Ji score									
Complete cases	99	15.1	9.5	19	17.3	9.3	118	15.4	9.43
Single imputation – mean score	105	14.9	9.3	23	16.3	8.8	128	15.2	9.22
Tung‐Chen score									
Complete cases	99	17.5	8.9	19	19.6	10.3	118	17.8	9.16
Single imputation – mean score	105	17.1	9.0	23	19.4	10.0	128	17.5	9.17

HDU, high‐dependency unit; ICU, intensive care unit.

**Figure 3:**
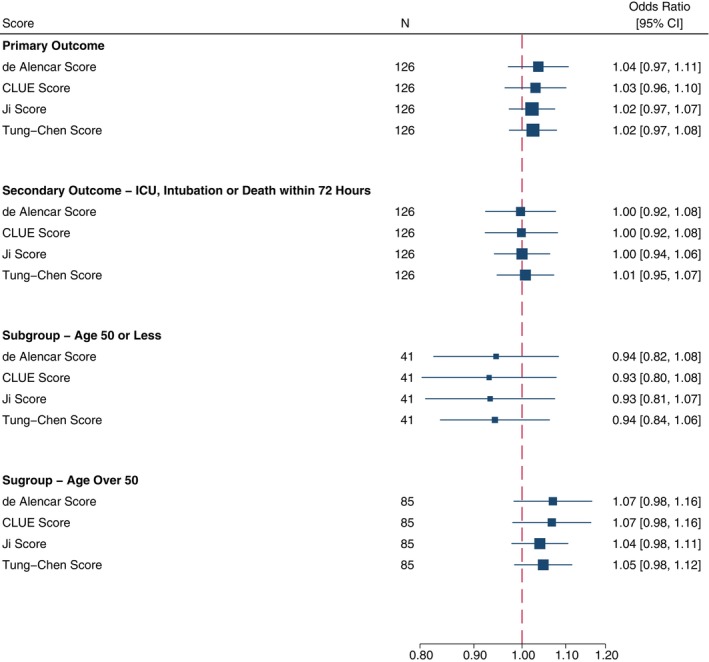
Odds ratio for logistic regression of ICU/HDU admission, intubation or death by lung ultrasound score. HDU, high‐dependency unit; ICU, intensive care unit.

Logistic regression analysis findings of the primary outcome by the LUS score are shown in Table 3. Following multiple imputation of missing data, no association was seen between increased the LUS score and the composite primary end point for the de Alencar score (OR = 1.04; 95% CI: 0.97–1.11; P = 0.29), the CLUE score (OR = 1.03; 95% CI: 0.96–1.10; P = 0.40), the Ji score (OR = 1.02; 95% CI: 0.97–1.07; P = 0.40) and the Tung‐Chen score (OR = 1.02; 95% CI: 0.97–1.08; Figure 3). Complete case and single imputation analyses showed similar results. Pseudo‐R^2^ for the complete case and single imputation analyses varied from a minimum of 0.004 (Ji score, single imputation analysis) to a maximum of 0.013 (de Alencar score, complete case analysis).

**Table 3:** Logistic regression of composite outcome of death, intubation or ICU/HDU admission by COVID‐19 lung ultrasound score; complete cases, simple imputation by mean score and multiple imputation by ordered logistic regression.

Score	N	Odds ratio	95% CI	P‐value	Pseudo‐R^2^
de Alencar score					
Complete cases	118	1.04	0.97–1.12	0.25	0.013
Single imputation – mean score	128	1.04	0.97–1.11	0.24	0.012
Multiple imputation – ordered logistic regression	126	1.04	0.97–1.11	0.29	–
CLUE score					
Complete cases	118	1.03	0.96–1.11	0.36	0.008
Single imputation – mean score	128	1.03	0.97–1.10	0.32	0.009
Multiple imputation – ordered logistic regression	126	1.03	0.96–1.10	0.40	–
Ji score					
Complete cases	118	1.03	0.97–1.08	0.34	0.009
Single imputation – mean score	128	1.02	0.97–1.07	0.51	0.004
Multiple imputation – ordered logistic regression	126	1.02	0.97–1.07	0.40	–
Tung‐Chen score					
Complete cases	118	1.03	0.97–1.08	0.34	0.009
Single imputation – mean score	128	1.03	0.98–1.08	0.27	0.010
Multiple imputation – ordered logistic regression	126	1.02	0.97–1.08	0.39	–

CI, confidence interval; HDU, high‐dependency unit; ICU, intensive care unit; N, number analysed.

Odds ratio reported for 1‐point increase in COVID‐19 lung score.

Nineteen participants met the primary composite outcome in complete case population, 24 in single imputation population and 23 in the multiple imputation population.

### Secondary outcomes

The secondary composite outcome of ICU admission, intubation and ventilation or death within 72 h of enrolment was met by 16 participants, with no association seen between this end point and the LUS scores (Table 4). No association was seen between the LUS score and the individual components of the primary outcome. A pre‐specified subgroup analysis by age category (age ≤ 50 or age > 50) showed no association between any LUS score and the primary outcome, for either the younger or older age category (Table 4).

**Table 4:** Secondary outcomes and subgroup analyses by COVID‐19 lung ultrasound score.

Score	N	Odds ratio	95% CI	P‐value
Secondary outcome – ICU/HDU admission from ED, intubation or death within 72 h.				
de Alencar score	126	1.00	0.92–1.08	0.93
CLUE score	126	1.00	0.92–1.08	0.97
Ji score	126	1.00	0.94–1.06	1.00
Tung‐Chen score	126	1.01	0.95–1.07	0.81
Secondary outcome – ICU/HDU admission from ED				
de Alencar score	126	0.99	0.92–1.07	0.82
CLUE score	126	0.99	0.92–1.08	0.88
Ji score	126	1.00	0.94–1.07	0.92
Tung‐Chen score	126	1.01	0.94–1.07	0.87
Secondary outcome – intubation				
de Alencar score	126	1.07	0.97–1.19	0.18
CLUE score	126	1.04	0.94–1.15	0.45
Ji score	126	1.03	0.96–1.11	0.45
Tung‐Chen score	126	1.03	0.95–1.11	0.52
Secondary outcome – death				
de Alencar score	126	1.06	0.95–1.18	0.27
CLUE score	126	1.09	0.97–1.22	0.15
Ji score	126	1.03	0.95–1.11	0.51
Tung‐Chen score	126	1.07	0.98–1.16	0.14
Subgroup analysis – age less than or equal to 50				
de Alencar score	41	0.94	0.82–1.08	0.42
CLUE score	41	0.93	0.80–1.08	0.34
Ji score	41	0.93	0.81–1.07	0.33
Tung‐Chen score	41	0.94	0.84–1.06	0.33
Subgroup analysis – age greater than 50				
de Alencar score	85	1.07	0.98–1.17	0.12
CLUE score	85	1.07	0.98–1.16	0.14
Ji score	85	1.04	0.98–1.10	0.21
Tung‐Chen score	85	1.05	0.98–1.11	0.17

CI, confidence interval; ED, emergency department; HDU, high‐dependency unit; ICU, intensive care unit; N, number analysed.

Odds ratio reported for 1‐point increase in COVID‐19 lung ultrasound score.

All secondary outcome and subgroup analyses performed following multiple imputation of missing lung zone data.

In the multiple imputation population, 15 participants met the secondary composite outcome of ICU/HDU admission from ED, intubation or death within 72 h, 13 participants met the secondary outcome of ICU/HDU admission from ED, 10 met the secondary outcome of intubation and 9 met the secondary outcome of death.

In the multiple imputation population, five participants met the primary outcome in the ‘Age less than or equal to 50’ subgroup. Eighteen met the primary outcome in the ‘Age greater than 50’ subgroup.

P‐value for interaction between the LUS score and ‘Age greater than 50’ – de Alencar score 0.13, CLUE score 0.11, Ji score 0.17 and Tung‐Chen score 0.12.

Higher levels of respiratory support at the time of enrolment were associated with higher LUS scores, including the de Alencar score (OR = 1.08; 95% CI: 1.03–1.13; P = 0.002), the CLUE score (OR = 1.09; 95% CI: 1.04–1.14; P = 0.001), the Ji score (OR = 1.06; 95% CI: 1.02–1.10; P = 0.002) and the Tung‐Chen score (OR = 1.05; 95% CI: 1.01–1.09; P = 0.007; Table 5). Higher estimated fraction of inspired oxygen at enrolment was also associated with higher de Alencar score [mean difference (MD) = 0.006; 95% CI: 0.001–0.011; P = 0.020], higher CLUE score (MD = 0.007; 95% CI: 0.002–0.012; P = 0.010), higher Ji score (MD = 0.004; 95% CI: 0.000–0.008; P = 0.037) and higher Tung‐Chen score (MD = 0.005; 95% CI: 0.001–0.009).

**Table 5:** Respiratory support and hospital length of stay by COVID‐19 lung ultrasound score.

Score	Analysis (measure of effect)	Point estimate^a^	95% CI	P‐value
Level of respiratory support^b^				
de Alencar score	Ordered logistic regression (odds ratio)	1.08	1.03–1.13	0.002
CLUE score		1.09	1.04–1.14	0.001
Ji score		1.06	1.02–1.10	0.002
Tung‐Chen score		1.05	1.01–1.09	0.007
Estimated fraction of inspired oxygen				
de Alencar score	Linear regression (mean difference)	0.006	0.001–0.011	0.020
CLUE score		0.007	0.002–0.012	0.010
Ji score		0.004	0.000–0.008	0.037
Tung‐Chen score		0.005	0.001–0.009	0.018
Length of stay^c^				
de Alencar score	Negative binomial regression (rate ratio)	1.06	1.02–1.08	<0.001
CLUE score		1.05	1.02–1.08	<0.001
Ji score		1.04	1.01–1.06	0.002
Tung‐Chen score		1.03	1.01–1.05	0.010

CI, confidence interval.

All secondary analyses performed following multiple imputation of missing lung zone data.

^a^ Point estimate for 1‐point change in COVID‐19 lung score.

^b^ Level of respiratory support: No supplemental O2, oxygen through nasal cannulae or oxygen through face mask and higher level support (including high‐flow nasal cannulae, non‐invasive ventilation, and intubation and ventilation.

^c^ Length of stay for patients who died during hospital admission analysed as the longest length of stay recorded (88 days).

Higher values of each of the studied LUS scores were associated with longer hospital length of stay. This included the de Alencar score [rate ratio (RR) = 1.06; 95% CI: 1.02–1.08; P < 0.001], the CLUE score (RR = 1.05; 95% CI: 1.02–1.08; P < 0.001), the Ji score (RR = 1.04; 95% CI: 1.01–1.06; P = 0.002) and the Tung‐Chen score (RR = 1.03; 95% CI: 1.01–1.05; P = 0.010; Table 5).

### Lung ultrasound scores

Pairwise Pearson's correlation coefficients for the four LUS scores are shown in Table 6. Pearson's correlation coefficient ranged between 0.82 and 0.97 for all pairs of the LUS scores, denoting a strong, positive correlation between scores.

**Table 6:** Pairwise Pearson correlation coefficients for lung ultrasound scores.

	de Alencar score	CLUE score	Ji score	Tung‐Chen score
de Alencar score	1.00	–	–	–
CLUE score	0.97	1.00	–	–
Ji score	0.92	0.89	1.00	–
Tung‐Chen score	0.91	0.95	0.82	1.00

All pairwise correlations statistically significant with P < 0.001, after Bonferroni adjustment for multiple comparisons.

Agreement by lung zone using linear weighted Kappa is displayed in Table 7. Substantial zone‐wise agreement was seen between the de Alencar and CLUE scores, with linear weighted Kappa of 0.90. The linear weighted Kappa statistic for other pairs of the LUS scores varied between 0.39 and 0.65, consistent with moderate agreement.

**Table 7:** Linear weighted Kappa statistic of agreement between lung ultrasound scores by zone.

	de Alencar score	CLUE score	Ji score^a^	Tung‐Chen score
de Alencar score	–	–	–	–
CLUE score	0.90	–	–	–
Ji* score	0.56	0.59	–	–
Tung‐Chen score	0.61	0.65	0.39	–

^a^ Lung component of Ji Score.

### Chest X‐ray and CT

Chest X‐ray and CT findings are summarised in Table 8. Chest X‐ray imaging was performed on a total of 127 of the recruited participants, whereas chest CT imaging was performed on 48 recruited participants. One or more abnormalities were found on chest X‐ray in 92% of participants, with the most common abnormalities being ground‐glass opacities (GGO; 57%) and consolidation (20%). No statistically significant associations were found between chest X‐ray findings and the primary outcome. One or more abnormalities were found on chest CT imaging in 98% of participants who underwent this modality, most commonly GGO (81%), consolidation (19%) and mediastinal lymphadenopathy (17%); an association was seen between bullous changes (0% vs. 40%; P < 0.001) and the primary outcome of intubation, ICU admission or death.

**Table 8:** Chest X‐ray and CT findings by composite outcome of death, ICU or HDU admission or intubation.

Findings on medical imaging	No death, ICU or HDU admission or intubation	Death, ICU or HDU admission or intubation	Overall	Odds ratio (95% CI)	P‐value
Chest X‐ray	N = 104	N = 23	N = 127		
Consolidation	22 (21%)	4 (17%)	26 (20%)	0.78 (0.24–2.54)	0.69
GGO	58 (56%)	14 (61%)	72 (57%)	1.23 (0.49–3.10)	0.66
Bilateral GGO	40 (38%)	11 (48%)	51 (40%)	1.47 (0.59–3.64)	0.41
Peripheral GGO	11 (11%)	4 (17%)	15 (12%)	1.78 (0.51–6.19)	0.36
Pleural effusion(s)	4 (4%)	1 (4%)	5 (4%)	1.14 (0.12–10.7)	0.91
Pulmonary nodules	3 (3%)	0 (0%)	3 (2%)	–	0.41
Lobar pneumonia	5 (5%)	0 (0%)	5 (4%)	–	0.28
Bullous changes	1 (1%)	0 (0%)	1 (1%)	–	0.63
Normal chest X‐ray	9 (9%)	1 (4%)	10 (8%)	0.48 (0.06–3.99)	0.49
Computed tomography	N = 43	N = 5	N = 48		
GGO	35 (81%)	4 (80%)	39 (81%)	0.91 (0.09–9.3)	0.94
Bilateral GGO	33 (77%)	4 (80%)	37 (77%)	1.21 (0.12–12.1)	0.87
Peripheral GGO	7 (16%)	0 (0%)	7 (15%)	–	0.33
‘Crazy‐paving’ pattern	2 (5%)	0 (0%)	2 (4%)	–	0.62
Consolidation	8 (19%)	1 (20%)	9 (19%)	1.10 (0.11–11.2)	0.94
Pleural effusion(s)	3 (7%)	0 (0%)	3 (6%)	–	0.54
Mediastinal lymphadenopathy	8 (19%)	0 (0%)	8 (17%)	–	0.29
Lobar pneumonia	1 (2%)	0 (0%)	1 (2%)	–	0.73
Bullous changes	0 (0%)	2 (40%)	2 (2%)	–	<0.001
Normal CT	1 (2%)	0 (0%)	1 (2%)	–	0.73

Data reported as number (percentage).

CI, confidence interval; CT, computed tomography; GGO, ground‐glass opacities; HDU, high‐dependency unit; ICU, intensive care unit.

No patients had pneumothorax or cavitation identified on chest X‐ray. No patients had subpleural GGO, bronchovascular thickening, traction bronchiectasis, multiple tiny pulmonary nodules, centrilobular nodules, pneumothorax or cavitation identified on CT.

## Discussion

In an international cohort of patients admitted from ED with COVID‐19, a representation of published LUS scores did not predict invasive ventilation, ICU admission or death. However, the different LUS scoring systems correlated reasonably well with each other, and scores were in keeping with other outcomes, such as the level of respiratory support and duration of hospital admission.

Invasive ventilation and death due to COVID‐19 is predominately related to the development of acute respiratory distress syndrome (ARDS), which typically occurs within a week of contracting the virus.^22^, ^23^ ARDS is characterised by diffuse alveolar damage, which manifests as hypoxia and bilateral infiltrates on chest imaging.^24^, ^25^ The LUS severity scores have been developed to quantify the extent of COVID‐19 disease and progression towards ARDS, with progressive loss of lung aeration.^6^, ^7^, ^8^, ^9^, ^26^ Therefore, the timing of LUS being performed during the patient's hospital journey is critically important for the purposes of prognostication.

Two studies performed LUS on patients admitted to hospital with COVID‐19 at around 1‐week postadmission that predicted invasive ventilation, ICU admission and death.^4^, ^8^ These were both single‐centre, prospective cohort studies, with Ji *et al*.^8^ recruiting 280 participants in Wuhan, China, in January to March 2020 with the LUS scores > 12/60 (12‐region protocol; lung and pleura findings) and Trias‐Sabria *et al*.^4^ recruiting 36 participants in Barcelona, Spain, in April 2020 with the LUS scores ≥ 24/36 (12‐region protocol), that predicted ICU admission, invasive ventilation and death. However, both of these studies may have been biased by being a selected cohort of admitted patients with known COVID‐19 with either well‐established pneumonia on non‐invasive respiratory support or admitted for other reasons with incidental mild disease. It is also unclear whether the addition of pleura findings adds value to the LUS severity score.^13^

In contrast, our cohort consisted of undifferentiated patients who presented to the ED with respiratory symptoms suggestive of potential COVID‐19 and were then admitted to hospital. By excluding patients who were discharged from the ED, we removed the potential bias from having an over‐representation of patients with mild disease and low scores. The multicentre, prospective cohort study by Sun *et al*.^27^ in February to March 2020 recruited 402 patients in Wuhan, China, who presented to ED with COVID‐19, had the LUS scores > 15/36 (12‐region protocol) that predicted mortality, but may have been biased by including patients who were discharged. Conversely, two studies were similar to our eligibility criteria, the single‐centre, prospective cohort study by de Alencar *et al*.^9^ from March to May 2020 demonstrated that 180 patients in Brazil, with the LUS scores > 18/36 (12‐region protocol), and the multicentre, prospective cohort study by Tung‐Chen *et al*.^7^ from March to September 2020 in Spain, with the LUS scores > 10, in combination with other markers, were both predictive of mortality.

Compared with these earlier studies performed at the start of the pandemic, the negative outcome of our study could reflect the changing scenario of the COVID‐19 pandemic, with the majority of patients recruited in early 2021. This could include factors such as higher rates of vaccination, higher innate immunity in the community, progressive weakening of the virulence of endemic COVID‐19 strains and the increasing availability of antiviral treatments.^28^, ^29^, ^30^ This was further supported in our data in that other imaging modalities, such as X‐ray and CT scans, were also not able to prognosticate outcomes.^31^ However, the LUS scoring systems did correlate with disease severity in terms of level of respiratory support, estimated fraction of inspired oxygen and hospital length of stay. These findings suggest that, although the LUS scores may accurately describe pathological lung changes, their prognostic value appears to be influenced by other patient, disease and system factors that contribute to these outcomes, which have evolved over time.

The initial study design intended a much larger sample size, to allow for an accurate estimate of the true prevalence of the composite end point. Unfortunately, the intended sample size was not achieved due to a lack of funding and increased clinical pressures associated with the COVID‐19 pandemic, raising the concern that the study may be underpowered to detect a true association. Exploratory *post hoc* power analysis was used to estimate the achieved power for a range of possible effect sizes, based upon the achieved sample size and the observed incidence of the primary outcome (Table S1). The analysis suggests that the study may have been underpowered to detect a weaker association between a LUS score and the primary outcome.

The main strength of our study was being an international, multicentre, prospective study, with comparison of several different LUS scores that were applied to the cohort. Multiple imputation was effectively used to impute missing data, and there was generally good agreement between the LUS scores and similar outcome findings, suggesting that our findings should hold for a range of score specifications. The weaknesses of our study included convenience sampling across a 2‐year period, which could have led to selection bias. The primary end point used in this study would not account for participants with severe COVID‐19 lung disease who were managed on the ward due to the ICU being overwhelmed. The low pseudo‐R^2^ values for the LUS scores in this study suggest that these scores are unlikely to be clinically important predictors of endotracheal intubation, ICU admission or death in a larger dataset, but the study was underpowered to draw any definite conclusion.

## Conclusions

In an international cohort of adult patients presenting to the ED with suspected COVID‐19 disease who had LUS performed and were subsequently admitted to hospital, four representative LUS severity scores did not prognosticate the composite outcome of invasive ventilation, ICU admission or death. However, the study did not reach the intended sample size and may have been underpowered to detect a statistical association between the LUS scores and the primary composite outcome, making it difficult to draw any firm conclusion. Although the study findings suggest that a clinically important relationship is unlikely, we cannot exclude the possibility that an effect may have been detected with a larger sample size. The LUS scores did correlate with the severity of COVID‐19 disease, including current respiratory support requirements and hospital length of stay, suggesting that the change in disease patterns and treatments may have cofounded the primary outcome. Further work is required to establish the use of the LUS scores in COVID‐19 disease prognostication, including the determination and prospective validation of a standardised scoring system, as well as the integration of these scores into management pathways for patients with pandemic respiratory viral disease.

## Author Contributions

**Peter J Snelling:** Conceptualization; writing – original draft; data curation; project administration. **Philip Jones:** Formal analysis; data curation; writing – review and editing. **Rory Connolly:** Conceptualization; writing – review and editing; data curation. **Tomislav Jelic:** Conceptualization; writing – review and editing; data curation. **Dan Mirsch:** Conceptualization; writing – review and editing; data curation. **Frank Myslik:** Conceptualization; writing – review and editing; data curation. **Luke Phillips:** Conceptualization; writing – review and editing; data curation; project administration. **Gabriel Blecher:** Conceptualization; writing – review and editing; data curation; project administration.

## Funding

No funding information is provided.

## Conflict of Interest

None to declare.

## Supporting information


**Table S1.** Exploratory power analysis.
