# Supplementary material for: Comparison of lung ultrasound scoring systems for the prognosis of COVID‐19 in the emergency department: An international prospective cohort study
Source: Australas J Ultrasound Med. 2023 Oct 29;27(2):75–88. doi: 10.1002/ajum.12364 (PMC11109992; doi:10.1002/ajum.12364)
Supplement: Supplementary file 1 — Table S1. Exploratory power analysis. [file AJUM-27-75-s001.docx]

**Supplementary Table 1:** Exploratory Power Analysis

| **Stage** | **Assumptions** | **Sample Size** | **Baseline Probability of Primary Outcome** | **Probability of Primary Outcome for Participant with mean + 1 SD LUS Score*** | **Power** |
| --- | --- | --- | --- | --- | --- |
| Before Data Collection | Planned sample size. Assumed baseline probability from prior studies. α = 0.05 | 1000 | 0.333 | 0.364 | 60% |
|  |  | 1000 | 0.333 | 0.374 | 80% |
|  |  | 1000 | 0.333 | 0.381 | 90% |
| After Data Collection | Achieved sample size. Estimated baseline probability from study data. α = 0.05 | 129 | 0.186 | 0.268 | 60% |
|  |  | 129 | 0.186 | 0.300 | 80% |
|  |  | 129 | 0.186 | 0.330 | 90% |
| SD: Standard Deviation  *Probability of composite primary outcome for participant with LUS score mean + 1 standard deviation that would allow study to achieve stated power. | | | | | |
